# Getting ready for integrated vector management for improved disease prevention in Zimbabwe: a focus on key policy issues to consider

**DOI:** 10.1186/s12936-019-2965-x

**Published:** 2019-09-23

**Authors:** Shadreck Sande, Moses Zimba, David Nyasvisvo, Munyaradzi Mukuzunga, Emmanuel H. Kooma, Joseph Mberikunashe, Busisani Dube

**Affiliations:** 1 Abt Associates Inc., Block 1 & 2 Westgate, Harare, Zimbabwe; 2 Department of Biological Science, University of Zimbabwe, Harare, Zimbabwe; 3 Ministry of Health and Child Care, Manicaland Directorate, Mutare, Zimbabwe; 4 National Malaria Elimination Centre, Chainama Hospital, Great East Road, Lusaka, Zambia; 5 Ministry of Health and Child Care, National Malaria Control Programme, Harare, Zimbabwe

**Keywords:** Integrated vector management, Vector-borne diseases, Vector control, Strategy, Zimbabwe

## Abstract

**Background:**

This paper outlines Zimbabwe’s potential readiness in harnessing integrated vector management (IVM) strategy for enhanced control of vector-borne diseases. The objective is to provide guidance for the country in the implementation of the national IVM strategy in order to make improvements required in thematic areas of need. The paper also assesses the existing opportunities and gaps to promote and adopt the approach as a national policy.

**Main text:**

Despite recent gains in combating vector-borne diseases, especially malaria, management of vector control programmes still remains insecticide-based and vertical in nature. Therefore, concerns have been raised on whether the current long-standing conventional vector control strategy still remains with sufficient action to continue to break the transmission cycle to the levels of elimination. This is so, given the continuous dwindling resources for vector control, changes in vector behaviour, the emergence of resistance to medicines and insecticides, climate change, environmental degradation, as well as diversity in ecology, breeding habitats, and community habits. Cognizant of all that, elements of a surveillance-driven IVM approach are rapidly needed to move vector control interventions a step further. These include advocacy, policy formulation, capacity building, public and private partnerships, community engagement, and increasingly basing decisions on local evidence. Understanding the existing opportunities and gaps, and the recognition that some elements of IVM are already imbedded in the current health programmes is important to encourage stakeholders to promptly support its implementation. Leveraging on the existing opportunities, combined with sufficient advocacy, IVM could easily be accepted by the Zimbabwe government as part of a wider integrated disease management strategy. The strategy could represent an excellent breakthrough to establish much needed intra and inter-sectoral dialogue, and coordination for improved vector-borne disease prevention.

**Conclusions:**

After synthesis of the opportunities and challenges clearly presented, it was concluded that it is imperative for Zimbabwe to adopt and implement IVM strategy that is informed by work already done, while addressing the bottlenecks. The significance of refocusing for improved disease prevention that has the potential to accomplish elimination of not only malaria but all vector borne diseases much earlier than anticipated under the existing vector control system is underscored.

## Background

The World Health Organization (WHO) [1, 2] intensified the campaign to countries for transforming the current long-standing conventional vector control systems to integrated vector management (IVM) strategy for improved vector-borne disease (VBD) control. Consequently, a renewed interest in the approach has emerged in some countries in sub-Saharan Africa [3]. The extent at which IVM has been adopted and promoted varies with countries. Some countries have embarked on consolidating strategic and operational frameworks. Whereas, others have gone a step further and adopted the strategy as a national policy, and have implemented its key elements with varying successes. The elements include enhanced advocacy, intra and inter-collaboration, integrated approach, capacity building, particularly human resource development, as well as basing decisions increasingly on local evidence, and community involvement and empowerment to ensure sustainability [3].

Defined as an approach to optimize and rationalize use of resources for vector control [2], IVM makes vector control more efficient, cost-effective, ecologically sound and sustainable. It integrates use of resources, targeting multiple VBDs, evidence-based use of chemical and non-chemical vector control tools, along with adaptive management [1, 2, 4]. By refocusing to IVM strategy, guided by the framework for a National Vector Control Needs Assessment (VCNA), vector control systems must better overcome the new challenges in the control of malaria and all VBDs in the continuous presence of managerial, financial, operational and infrastructure deficiencies [1].

Several VBDs commonly coexist in the same ecosystems, impact negatively to the affected human populations, especially in developing countries. The VBDs contribute substantially to global disease burden and disproportionately affect communities [1, 2]. The key vector-borne diseases being malaria, lymphatic filariasis, dengue fever, cutaneous leishmaniasis, visceral leishmaniasis, onchocerciasis, human African trypanosomiasis (HAT) and schistosomiasis. In response, vector control has proven to be an important component to significantly interrupt transmission. This has been shown convincingly over the years in regions where malaria has been eliminated [1].

In Zimbabwe, vector control has been the most widely used malarial control intervention. The strategy has been linked with reduction in disease burden in recent years [5]. Cognizant of such a drastic decrease in cases in several areas across the country [5–7], Zimbabwe’s National Malaria Control Programme (NMCP) selected some regions for programme reorientation and implementation of malaria elimination strategies [5].

Even with this well-documented evidence showing significant progress over the years, concerns have been raised whether or not the current long-standing conventional vector control systems must remain with sufficient action to continue to break the disease transmission cycle to the levels of elimination. This is so, given the new challenges; emerging and re-emerging of vectors, changes in vector behaviour, resistance to medicines and insecticides, use of a single tool, control of a single VBD, climate change, dam construction, irrigation projects, environmental degradation, urbanization, human resettlements, increased cross-border population movements, as well as diversity in ecology, breeding habitats, and community habits [2].

For maximum benefits, current challenges for vector control interventions may need new tools and different approaches for improved disease management and sustainability. In light of all that, the aim of this paper is not to revisit the elements and processes of IVM, but rather, to clearly show its growing need, as well as to contribute and provide the much needed guidance and confidence to stakeholders and implementers through evidently pinpointing work already done and current challenges that require attention. Also, to highlight some fundamentals to be linked-up that Zimbabwe can leverage on to move not only towards malaria elimination but to all VBDs earlier than previously anticipated. Therefore, it is imperative that Zimbabwe adopts IVM before implementing the WHO Global Vector Control Response (GVCR), a second generation strategy that builds on the basic concepts of IVM approach, aimed with renewed focus on improved human capacity at national and subnational levels, and infrastructure strengthening [8].

## Policy and regulatory frameworks

Most key policy and regulatory frameworks to manage health delivery system are available, dating back to the early 1980s (Table 1).

**Table 1:** Zimbabwe Health Policies and Acts from 1984 to 2020 including International Health Regulations, 2005

Policy/Act of Parliament	Period	Lead Ministry (Sector)	Achievement
Planning for equity in health	1984	MoHCC	Health imbalances and contemporary health needs addressed by promoting universal access to antimalarials, IRS and LLINs to treat and prevent malaria
The National Health Strategy for Zimbabwe “Working for Quality and Equity in Health”	1999–2007		
The National Health Strategy for Zimbabwe “Equity and Quality in Health: a people’s right”	2009–2013		
The National Health Strategy for Zimbabwe “Equity and Quality in Health: leaving no one behind”	2016–2020		
Zimbabwe Malaria Business plan	2014–2017	MoHCC (NMCP)	Mobilization of resources for malaria control enhanced
Malaria Communication Strategy	2016–2020	MoHCC (NMCP)	Framework for planning, implementation, monitoring and evaluation of malaria communication programmes at all levels improved
Health Services Act (Chapter 15;16)	2004	MoHCC	Health committees existing at different levels of the health delivery system created, community participation in policy development and decision making process strengthened
Public Health Act (Chapter 15:17)	2018	MoHCC	Linkage between preventive and curative health care interventions strengthened
Environmental Management Act (Chapter 20:27)	2005	METHI (EMA)	The much needed protection to flora and fauna enhanced through control of transportation and management of insecticides for public health, agriculture and household use
Animal Health Act (Chapter 19:01)	2001	MLAWCRR (TCD)	The platform for the prevention and elimination of the species of tsetse fly that transmit trypanosomiasis to both humans and livestock provided
Income Tax Act (Chapter 23:06)	2004	MFED (ZIMRA)	Quantities of imported anti-malarials, insecticides and all other related vector control commodities increased following the exemption of tariffs and taxes
International Health Regulation	2005	WHO-Member States	Integrated disease surveillance across international borders strengthened

*MoHCC* Ministry of Health and Child Care, *IRS* indoor residual spraying, *LLINs* long-lasting insecticidal nets, *METHI* Ministry of Environment, Tourism and Hospitality Industry, *EMA* Environmental Management Agency, *MLAWCRR* Ministry of Lands, Agriculture, Water, Climate and Rural Resettlement, *TCD* Tsetse Control Division, *MFED* Ministry of Finance and Economic Development, *ZIMRA* Zimbabwe Revenue Authority

These strategic policy documents are administered by various divisions independently. The implementation or non-implementation by one entity affects the others. In addition, the health service delivery system has been also controlled by general acts of parliament policy documents that are suitable legal and regulatory frameworks (Table 1). Also, a political will exists in the country as demonstrated by establishment of a national health regulatory authority that controls the importation of insecticides. The registration process of all imported insecticides for public health use has been strictly controlled, involving a two-phase evaluation and testing scheme. This includes laboratory testing procedures conducted by the Department of Agricultural, Technical and Extension Services under MLAWCRR, as well as field evaluation by the National Institute of Health Research, a Department of MoHCC. The government’s commitment to exempt tariffs and taxes on insecticides and all other related vector control commodities and supplies [9] further illustrates its strong political will to support disease control interventions.

Although the country has made important strides to develop these strategic policy documents to properly guide the health service delivery system, most of them appear to be silent on promotion of IVM, focusing predominantly on malaria prevention. While schistosomiasis and HAT are mentioned in the current National Health Strategy (2016–2020), lack of details for the vector-borne diseases in the control strategy prompts suggestions that the current governing health regulatory policy frameworks are used largely against malaria as a single disease.

There has been also a shortage of these policy documents at the periphery level of service provision, and supervision of their implementation being limited at all levels. The country’s lack of regulations and close supervision to change irrigation practices to ensure mandatory vegetation clearance, flushing or regular drying of canals to limit breeding habitats for vectors that cause malaria, schistosomiasis and other VBDs clearly illustrates this gap.

Therefore, it remains clear that the work already done on policy and regulatory frameworks have gaps that require attention. To jointly own and administer policy and regulatory frameworks currently under different entities (Table 1), adoption and implementation of IVM must be an asset. This must provide a glaring opportunity to include all other VBDs in later versions of policy documents, as well as development of additional guidelines and standards such as National IVM policy that must help to strengthen collaboration at national through to village level. Similarly, the strategy must strengthen cross-sector efforts and accountability among major partners such as agriculture, education, finance, public works, local government, mines, environmental management agency, non-governmental organization (NGOs), civil society, academic research, and community whose actions/inactions contribute considerably to local disease burdens.

## Technical, financial and infrastructure development

Currently, Zimbabwe has approximately 15 Medical Entomologists working in government ministries, research institutions, universities and NGOs (Mberikunashe, unpublished data). Each of the 59 rural administrative districts has at least one Environmental Health Officer (EHO), with 59% of the 1569 rural administrative wards being managed by Environmental Health Technicians (EHTs). Sixty percent of approximately 9414 villages have access to a Village Health Worker (VHW) [10]. Village Health Workers are community-based health aides selected, trained and working in the villages which they reside [11]. Of the EHOs and EHTs in post, 28% (275/985) have attended non-certificate training on basic entomology for malaria vector control (Mberikunashe, unpublished data). In close liaison with national level, these officers support entomological monitoring at local levels, particularly malaria vectors. The entomological work is complemented by the efforts of VHWs, whose basic knowledge on entomology is centred largely on field ad-hoc training. To be successful, an entomological monitoring programme must have appropriate infrastructures, especially laboratories, insectaries and field sentinel sites. In Zimbabwe there are three laboratories, three insectaries and 20 field sentinel sites that are functional and open for use by malaria partners to mainly monitor malaria vectors. Of these, two laboratories and two insectaries, and all sentinel sites are owned by government, with the remainder being a private entity. For tsetse fly monitoring, there are two laboratories and one insectary managed by TCD under Ministry of Agriculture.

Adequate infrastructure and trained human resources require sufficient funding that can be better mobilized from multiple partners. Financial contributions by different agencies for the past 3 years (2016–2018) for malaria prevention are shown on Table 2. Budget allocations for 2018 for most of the funding agencies were not readily accessible.

**Table 2:** Approximate financial contributions by major malaria partners in US dollar from 2016 to 2018, excluding human resources, equipment and infrastructure development

Partner	2016	2017	2018
GoZ	1,000,000	950,000	1,000,000
Global Fund	21,823,373	15,460,784	13,627,866
USAID/PMI	18,147,536	15,120,000	13,500,000
Bill Melinda Gates	218,417	224,970	–
United Methodist Church	270,000	300,000	–
Tongaat Hulett (Private company)	364,105	364,105	–
WHO	67,180	42,685	–
Total	41,890,611	32,462,544	28,127,866

*GoZ* Government of Zimbabwe

Although there has been an overall decrease in the programme budget between years, commitment to prevent and control malaria has been clearly evident for local and international funding agencies. The Global Fund to Fight AIDS, Tuberculosis and Malaria (GFATM) was the major contributor to malaria budget allocation for Zimbabwe for the entire 3-year period. From the GFATM budget, vector control got the largest share (Table 3), perfectly illustrating partners’ commitment to control vectors of public health importance.

**Table 3:** Percentage allocation of Global Fund malaria budget by thematic area 2018–2020

Thematic area	Budget allocation (US$)	%
Vector Control	33,010,111	64
Case Management	5,915,672	11
Specific Prevention Interventions	487,328	1
Health Management Information Systems, Monitoring and Evaluation	3,606,018	7
Programme Management	8,666,648	17
Grant total	51,685,777	100

The decision to shift from vector control to IVM has been to some extent motivated by already available in-country technical, financial and infrastructure capacities. Historically, the implementation of IVM approach has been slow and complicated due to several reasons, chief being over-estimation of the resource requirements, particularly finances. Little was known then, that IVM could in principle, be adopted without much additional resources [12]. The IVM approach is an organizational strategy designed to re-orient exiting systems, including resources to make them more cost-effective and efficient [1].

A good example being the Zambian experience where malaria control and elimination programme shifted from traditional vector control interventions to IVM strategy using malaria grant from GFATM [13]. Learning from the Zambian experience, Zimbabwe can also use the current grant from the GFATM (Table 3) to successfully introduce IVM to strengthen disease control interventions.

Even though, financial support for 2019 moving forward from all malaria control partners was not readily available, assuming the observed trends over the past 3 years (Table 2) continue, these funds could also be harnessed to complement the GFATM grant and used under the umbrella of IVM for improved disease management. The GoZ has to make a bold decision on which ministry the IVM programme must follow under as its success largely depends on the political will to drive the agenda and influence implementation.

To maximize the benefits of IVM, there has been a need to strengthen laboratory equipment and consumable supplies, as well as enhancing the knowledge and skills of personnel in the laboratories and sentinel sites beyond malaria vectors. Also, the current training on entomology for EHOs, EHTs and VHWs must be more formal and strengthened by following the WHO training guidelines on IVM [14]. The training guidelines must be appropriately modified not only to address the local needs but to ensure their appropriateness to each category of participants. Building this capacity in the vector control interventions has been an ongoing process and must occur from central to local levels [13], each training session must be informed by past experience and lessons learned from both class and field.

## Vector control methods

Zimbabwe has been a host to some vectors that cause endemic VBDs, including neglected tropical diseases (Table 4). Data on trypanosomiasis have been limited over the years. As explained by Shereni et al. [23], most probably, the spatial distribution and relative abundance of the two species of tsetse fly known to transmit the disease to both humans and livestock in the country have not been extensively studied. Similarly, the relatively low number of reported cases of HAT in the entire decade could be due to limited diagnostic capacity for the disease in most national health facilities, leading to gross under detection, significantly concealing the true epidemiological pattern of the disease.

**Table 4:** Control of endemic VBDs in Zimbabwe

Disease	Parasite	Vector	Distribution	Burden	Control measure	Lead sector	Refs.
Malaria	*Plasmodium falciparum, P. ovale, P. malariae*	*Anopheles arabiensis, An. gambiae, An. coluzzii, An. funestus*	Nationwide	470,000 cases (2017)	Case management, IRS, LLINs, SBCC	NMCP	[15–18]
Schistosomiasis	*Schistosoma haematobium, S. mansoni*	*Biomphalaria pfeifferi, Bulinus globosus*	Nationwide	3,255,067 cases (2014)	Treatment, MDA, snail control, WASH	EDC	[19–22]
HAT	*Trypanosoma brucei,* sub-species *T. b. rhodesiense*	Savanna group*, Glossina morsitans, G. pallidipes*	Mainly restricted to the northern districts along the Zambezi valley	28 cases (2005–2015)	Ground spraying, aerial spraying, cattle dipping, insecticide-treated odour-baited targets	TCD	[23]

*SBCC* social and behaviour change communication, *MDA* Mass drug administration, *WASH* water, sanitation and hygiene, *EDC* epidemiology and disease control

However, in response to the occurrence of VBDs, the country has implemented different vector control strategies that are managed by various sectors. Yet, management of most of the VBD control programmes still remains vertical in nature, targeting a single disease in most instances. To explore synergies and maximize the efforts of various sectors on vector control, there has been a need for the integration of VBD control by adoption of IVM strategy. Integrated approach must ensure rational use of available resources through a multi-disease control strategy, an example being combining chemical and non-chemical vector control tools, where appropriate, for enhanced disease prevention.

Even with clear guidelines on IVM [1, 2], Zimbabwe continues to be skeptical about the strategy, hence the delay in its introduction and implementation. As explained by Beier et al. [12], one of the major reasons for the skepticism that has caused some delays in the promotion of IVM is due to over-sophistication of integrated control by countries. Informed by work already done in Zimbabwe (Table 4), enough has been known already to base the adoption and implementation of integrated control.

## Partnerships and networks

Formal collaboration between health and other organizations such as public, private, and civil sectors, as well as communities have already partly been established under the leadership and coordination of various sectors (Table 5).

**Table 5:** Major committees of public health importance by operational level

Key committee	Partner	Core function	Frequency
National			
Malaria case management	NMCP^a^, ZAPIM, PMI, WHO, PMD representatives, PSI, CHAI	Development of malaria case management policies and guidelines	Quarterly
Vector control	NMCP^a^, VL, PMI, WHO, ZAPIM, Plan International, CHAI, PMD representatives, academia, researchers	Development of vector control policies and guidelines	Quarterly
POPs	METHI (DENR^a^), EMA, NMCP, City Health, parastatals, public laboratories, national insecticide registry, civil society, academia, researchers, experts	Controlling the use of POPs including pesticides and industrial chemicals to reduce devastating effects in flora and fauna resulting from contamination of the environment and food	Bi-annually
Trypanosomiasis control	TCD^a^, EDC, FAO, academia, researchers, experts	Development of policies on trypanosomiasis control, tsetse fly control	Bi-annually
Provincial			
PHT	MoHCC^a^, RDCs, NGOs, religious organizations	Development and implementation of provincial annual plans on health	Quarterly
PD	MLG^a^, All ministries, RDCs, parastatals	Management of developmental projects in the provinces including health	Monthly
District			
DHT	MoHCC^a^, rural health centres, mission hospitals, RDCs, NGOs	Development and implementation of district annual plans on health	Quarterly
DD	MLG^a^, MoHCC, All ministries, RDCs, parastatals	Management of developmental projects in the districts including health	Monthly
Ward/village			
Ward Health	MoHCC^a^, VHWs, SHCs, councilors, religious leaders, local community leaders	Planning and implementation of local health programmes, community-based management of malaria by VHWs	Monthly

*ZAPIM* Zimbabwe assistance programme in malaria, *PMD* Provincial Medical Director, *VL* vector link, *POPs* persistent organic pollutants, *DENR* Department of Environment and Natural Resources, *PHT* Provincial Health Team, *RDCs* rural district councils, *PD* provincial development, *MLG* Ministry of Local Government, *DHT* District Health Team, *DD* district development, *SHCs* School Health Coordinators

^a^Major stakeholder

Membership, core functions and frequency of scheduled meetings vary across committees. The majority of the members of most of the health committees at all levels are drawn from the MoHCC and health professionals from other sectors, while other committees have a wider membership integrating health and non-health experts.

However, the agenda items for the health committees are strictly health-related subjects and disease-specific, whereas other committees discuss developmental projects including health. The health committees at peripheral levels facilitate the engagement of local communities that actively participate in several health programmes including IRS, LLINs, larval source management (LSM) for malaria control, and selection of community volunteers appropriate to participate in various health programmes at local level.

Despite the existence of committees at all levels, networking on VBDs to coordinate action and enable effective sharing of information and resources has been relatively weak, with a higher chance of duplication of topics. Given all that, with adaptive management and reorientation, these committees must be used as vital communication channels not only to facilitate a smooth shift from the current system of vector control to IVM, but must as well bring success to the implementation of the strategy.

To standardize and facilitate a coherent approach, some current national committees must combine to form a national IVM steering committee. This committee must oversee, coordinate, review policies, mobilize resources, conduct quality control and quality assurance, conduct operational research, strengthen community involvement, ownership and empowerment, and guide the deliberations of other groups at lower levels. An efficient, cost-effective, ecologically sound and sustainable vector control programme to benefit the majority of Zimbabweans must be accomplished through enhanced combined efforts and collaboration among sectors.

## Research and development

Zimbabwe continues to build the evidence base to strengthen the understanding of localities where current vector control tools work best, and to determine how individual methods are affected by change in vector bionomics, ecosystems and socioeconomic settings, and search for new tools. The study conducted at Africa University in Zimbabwe to evaluate repellent properties of a permethrin-treated blanket exemplifies this [24]. The results showed a significant protection from mosquito bites, suggesting that with a wider promotion of the tool, it could be a new vector control strategy to complement the current vector control tools.

Major malaria vector samples of *Anopheles funestus* collected from both Mutare and Mutasa Districts in Zimbabwe, showed resistance to deltamethrin and lambda-cyhalothrin (pyrethroids), and bendiocarb (carbamate), but were susceptible to DDT (organochlorine) and pirimiphos-methyl (organophosphate) [25]. As deltamethrin and lambda-cyhalothrin were used interchangeably for IRS to control malaria transmission in both study sites, Zimbabwe’s NMCP, immediately switched to pirimiphos-methyl. Similarly, work by Kanyangarara et al. [6], observed reduction in malaria incidence following IRS campaign with pirimiphos-methyl in a setting with evidence of pyrethroid resistance in Mutasa District, Zimbabwe. Informed by this study, the NMCP has strengthened its implementation of insecticide resistance management plan.

The decision to implement a nationwide MDA to control schistosomiasis among the Zimbabwean populations was informed by a recent survey on schistosomiasis and soil transmitted helminthiasis [21]. Cognizant of the recommendations from this study, the implementation of MDA has been divided into two cohorts. One cohort encompasses uninterrupted annual MDA in districts where the prevalence of schistosomiasis is ≥ 50%. The other group involves annual MDA in districts where the prevalence of heavy infection by any schistosome species is ≥ 10%.

Shereni et al. [23], emphasized the importance of collecting and analysing spatially-explicit information on HAT, including entomological and parasitological data for evidence-based decision making to improve control interventions. Also, stressed in the same survey, is the need for coordinated multi-national actions involving Mozambique, Zambia and Zimbabwe, given the transboundary nature of the vector and the disease.

Elsewhere in Africa, the combination of various vector control strategies was also supported by findings of Maheu-Giroux and Castro [26] that exhibited significant synergist effects between larviciding and use of mosquito nets, window screens, and houses with a complete ceiling. Indeed, the results of local and international studies present a clear opportunity for Zimbabwe’s NMCP to ride on, and adopt IVM as a key strategy for improved vector-borne disease prevention. However, more evidence-based data at implementation levels are needed on the selection of single or multiple tools to prevent single or multiple vector-borne diseases.

## Conclusions

Opportunities and potential readiness to adopt, implement and manage IVM strategy exist and is great in Zimbabwe. The existing public health policy and regulatory frameworks, technical, financial and infrastructure capacities, partnerships and networks, as well as knowledge on occurrence of VBDs in the country and use of evidence for decision making, present clear prospects for adopting IVM for enhanced disease control in Zimbabwe.

Insights presented in this paper are to gently remind stakeholders that the major elements of IVM mentioned are already being implemented within the current health programmes and are meant to convey a perfect communication to ensure prompt promotion and adoption of the strategy. However, understanding work already done towards IVM does not provide justification to ignore key challenges highlighted that have the abilities to critically hold back the success of the strategy.

Weak advocacy, inadequate promotion of IVM, weak inter-sectoral and inter-programmatic strategic policy and regulatory frameworks, weak network to coordinate VBDs and sharing of information and resources are some of the challenges likely to threaten IVM strategy. Also, the success of the strategy could be threatened by inadequate implementation of a multi-disease approach as an integral part of disease control, along with less coordinated research agenda.

After synthesis of the opportunities and challenges clearly presented, it was concluded that it is imperative for the NMCP and partners should conduct enhanced advocacy for Zimbabwe to adopt and implement IVM strategy that has been informed by work already done, while addressing the bottlenecks. The significance of refocusing for improved disease prevention that has the potential to accomplish elimination of not only malaria but all VBDs much earlier than anticipated under the existing vector control system is underscored.

## Data Availability

Information was sourced from published and unpublished materials and the supporting data are included in the context.

## Abbreviations

CHAI: Clinton Health Access Initiative
DD: district development
DDT: dichlorodiphenyltrichloroethane
DENR: Department of Environment and Natural Resources
DHT: District Health Team
DLVS: Department of Live Stock and Veterinary Services
EDC: epidemiology and disease control
EHO: Environmental Health Officer
EHT: Environmental Health Technician
EMA: Environmental Management Agency
FAO: Food and Agriculture Organization
GFATM: Global Fund to Fight AIDS, Tuberculosis and Malaria
GVCR: Global vector control response
GoZ: Government of Zimbabwe
HAT: human African trypanosomiasis
IRS: indoor residual spraying
IVM: integrated vector management
LLINs: long-lasting insecticidal nets
LSM: larval source management
METHI: Ministry of Environment, Tourism and Hospitality Industry
MFED: Ministry of Finance and Economic Development
MLAWCRR: Ministry of Lands, Agriculture, Water, Climate and Rural Resettlement
MLG: Ministry of Local Government
MoHCC: Ministry of Health and Child Care
NGO: non-governmental organization
NMCP: National Malaria Control Programme
PD: provincial development
PHT: Provincial Health Team
PMD: Provincial Medical Director
PMI: President’s Malaria Initiative
POPs: persistent organic pollutants
PSI: Population Services International
RDCs: rural district councils
SBCC: social and behaviour change communication
TCD: Tsetse Control Division
USAID: United States Urgency for International Development
VBDs: vector-borne diseases
VHW: Village Health Worker
VL: vector link
WASH: water, sanitation and hygiene
WHO: World Health Organization
ZAPIM: Zimbabwe assistance programme in malaria
ZIMRA: Zimbabwe Revenue Authority
